# Increased Prevalence of Attention Deficit Hyperactivity Disorder in Individuals with Selective Immunoglobulin A Deficiency: A Nationwide Case–Control Study

**DOI:** 10.3390/jcm13206075

**Published:** 2024-10-12

**Authors:** Eugene Merzon, Reem Farag, Shai Ashkenazi, Eli Magen, Iris Manor, Abraham Weizman, Ilan Green, Avivit Golan-Cohen, Akim Genshin, Shlomo Vinker, Ariel Israel

**Affiliations:** 1 Adelson School of Medicine, Ariel University, Ariel 4070000, Israel; reemfaragam@gmail.com (R.F.); shaias@ariel.ac.il (S.A.); 2 Leumit Health Services, Tel Aviv-Yafo 6473817, Israel; igreen@leumit.co.il (I.G.); agolanchoen@leumit.co.il (A.G.-C.); ageyshin@leumit.co.il (A.G.); svinker@leumit.co.il (S.V.); aisrael@leumit.co.il (A.I.); 3 Department of Medicine A, Assuta Ashdod University Hospital and Faculty of Health Sciences, Ben-Gurion University, Beer-Sheba 8410501, Israel; allergologycom@gmail.com; 4 Department of Psychiatry, Faculty of Medicine, Tel Aviv University, Tel Aviv 6997801, Israel; dr.iris.manor@gmail.com (I.M.); weizmana@gmail.com (A.W.); 5 ADHD Unit, Geha Mental Health Center, Petah Tikva 4910002, Israel; 6 Department of Family Medicine, Faculty of Medicine, Tel Aviv University, Tel Aviv 6997801, Israel; 7 Department of Epidemiology and Disease Prevention, Faculty of Medicine, Tel Aviv University, Tel Aviv 6997801, Israel

**Keywords:** ADHD, immunoglobulin A, immune deficiency, secretory antibodies, infections, neurodevelopmental disorders, methylphenidate

## Abstract

**Background**: Selective Immunoglobulin A Deficiency (SIgAD) is one of the most prevalent immunodeficiencies, characterized by an increased risk of mucosal infections. Attention deficit hyperactivity disorder (ADHD) is among the most common neurodevelopmental disorders and is associated with significantly higher rates of various infectious diseases, white blood cell abnormalities, and considerable morbidity. This study aimed to evaluate the prevalence of ADHD among patients with SIgAD. **Methods**: We conducted a retrospective, observational, population-based case–control study, within Leumit Health Services, by comparing individuals diagnosed with SIgAD to a matched control group. Data were extracted from electronic health records. **Results**: Of the >700,000 registered individuals, 772 aged ≥4 years with SIgAD were identified (mean age 22.0 ± 17.5 years; male/female ratio 1:1). The 5:1 matched control group consisted of 3860 subjects without SIgAD, with no significant differences between the groups regarding age, gender, ethnicity, and socioeconomic status. ADHD prevalence was significantly higher in the SIgAD group (16.2%) than in the control group (12.9%), with an odds ratio of 1.30 (95% confidence interval 1.05–1.61, *p* = 0.017), as was the use of methylphenidate (6.6% vs. 4%). Additionally, respiratory and intestinal infections were significantly more common in the SIgAD group (*p* < 0.001). **Conclusion**: A significantly higher prevalence of ADHD was observed in patients with SIgAD compared to strictly matched controls without SIgAD. These findings enhance our understanding of the pathophysiology of ADHD and its associated health complications.

## 1. Introduction

### 1.1. Selective Immunoglobulin A Deficiency

Selective Immunoglobulin A Deficiency (SIgAD) has been recognized as the most prevalent congenital immunodeficiency, with an incidence ranging from 1:14 to 1:96 and affecting both genders equally [1]. SIgAD is characterized by a decreased or absent serum level of serum IgA, typically defined as a serum IgA level of less than 7 mg/dL while maintaining normal levels of the other immunoglobulin isotypes. Although many individuals with SIgAD may remain asymptomatic, others exhibit an increased susceptibility to infections, particularly mucosal sinopulmonary infections [2]. Clinical complications of SIgAD include an elevated risk of respiratory infections caused by bacteria and viruses, including COVID-19 [3], gastrointestinal infections with a particular susceptibility to *Giardia lamblia*, as well as a myriad of autoimmune diseases such as systemic lupus erythematosus, hyperthyroidism, hypothyroidism, type 1 diabetes, celiac disease, and also malignancies [1,2,4]. In addition, neurologic disorders, such as epilepsy, autism spectrum disorders, and tics, are more common in patients with SIgAD than in matched controls [5].

### 1.2. Attention Deficit Hyperactivity Disorder

Attention deficit hyperactivity disorder (ADHD) is one of the most common neurodevelopmental disorders; its prevalence in children and adolescents ranges between 5.9% and 9.5% and in adults between 2.5% and 2.8%; it is more common in males than females [6]. Multiple studies have shown that ADHD stems from a combination of polygenetic, neurobiological, and environmental factors, and is related to inflammatory processes [6]. Recent studies have highlighted associations between ADHD and diverse types of infections [3,7,8,9], autoimmune conditions [10,11,12], and heightened neuroimmune responses to infection and inflammation [13,14,15]. For example, our previous study indicated that children with ADHD experienced higher rates of multiple childhood infections, such as upper respiratory tract infections, acute gastroenteritis, and dermatomycosis, as well as higher rates of consumption of various antimicrobial agents, compared to children without ADHD [9]. Additionally, a recent study of our group demonstrated also that ADHD was associated with a more severe course of COVID-19 [13].

A recent large population-based study encompassing more than 2.5 million German youths revealed that individuals with ADHD were four times more likely to exhibit white blood cell abnormalities [16]. Another research revealed a significant positive association between ADHD and the anti-Yo antibody immunoreactivity in the Purkinje cells of the cerebellum, suggesting immune dysregulation in children with ADHD [17].

### 1.3. Aims of Study

The association between SIgAD and infectious diseases and autoimmunity and the association between ADHD and infections and inflammation, as detailed above, raise the question of a possible link between SIgAD and ADHD. This query prompted us to undertake the present study to explore the association of SIgAD and ADHD in a large-scale study. Such an association is important for physicians who take care of patients with SIgAD and will shed further light on the pathophysiologic relations between immunity, infections, and neurodevelopmental disorders.

## 2. Materials and Methods

### 2.1. Study Design

We conducted a population-based case–control study. For each patient with SIgAD, five strictly matched individuals without SIgAD (controls) were selected and compared for multiple associations. The study was performed among members of Leumit Health Services (LHS), a large, nationwide health maintenance organization (HMO) in Israel, which provides services to more than 700,000 members. LHS has a comprehensive computerized database that is continuously updated regarding demographics, medical diagnostic codes based on the International Classification of Diseases, 9th Revision, Clinical Modification (ICD-9-CM), and laboratory tests performed at a single centralized laboratory. The database includes also the medication prescribed for the registered subjects, and confirmation of medication purchase by the individual patients. All LHS members have similar general health insurance and equal access to the health services. The validity of the registry has been previously examined and confirmed as high [18]. The study was approved by the Institutional Review Board for Clinical Studies of LHS (approval number LEU 0005-22).

### 2.2. Study Population

The inclusion criteria of the study group included individuals aged ≥4 years who were registered at LHS from March 2020 through November 2022. Age below 4 years was an exclusion criterion, as ADHD is seldom diagnosed before the age of 4 years. The cases and control groups were identified from the study population as defined below. The demographic, clinical, and laboratory data of individuals were extracted from the computerized database, as were the diagnoses (based on ICD-9-CM codes) and medication purchased.

### 2.3. Definitions

For the cases group, i.e., to identify patients with SIgAD, we searched the electronic database of LHS for individuals aged ≥4 years with a diagnosis of SIgAD (ICD-9-CM code 279.01) and those with serum IgA levels measured by multiplex immunoassay (Bio-Rad, Hercules, CA, USA) less than 7 mg/dL. Patients were excluded from the study if one or more of the following chronic diagnoses were documented in the electronic health record: infection with human immunodeficiency virus, common variable immunodeficiency, severe combined immunodeficiency, ataxia-telangiectasia, DiGeorge syndrome, agammaglobulinemia, or other diagnosis of immunodeficiency.

The control group included subjects without SIgAD from the population registered at LHS during the same study period, with a case–control ratio of 1:5. Each subject in the control group was precisely matched to a case subject based on age category (at 10-year intervals), gender, ethnic sector, socioeconomic status (SES) category, and year of initial LHS membership, to minimize the potential confounding effects. Missing SES data (0.26%) were categorized separately for matching purposes. For each case, the five control subjects with the closest birth dates to the case’s birth date were selected. A post hoc comparison of the main demographic variables was performed to verify similar distributions in the cases and control groups.

ADHD was diagnosed according to the Israeli Ministry of Health requirements, which follows international guidelines. The diagnosing physician must be a senior one who has a specialty in the ADHD field, such as child or adult psychiatrists, child or adult neurologists, or pediatricians and family physicians with certified ADHD training. The diagnosis is established according to the American Psychiatric Association’s Diagnostic and Statistical Manual [19].

The SES was defined by the Israeli Central Bureau of Statistics classification of 10 categories. Individuals in categories 1 to 3 were defined as low SES, 4 to 6 as middle SES, and 7 to 10 as middle–upper SES. The ethnicity of the health maintenance organization member was categorized into three sectors: the general population, Ultra-Orthodox Jews, and Arabs. The latter two sectors have been documented by large-scale epidemiologic studies as having significantly higher rates of primary immunodeficiencies and infections than the rest of the Israeli population.

### 2.4. Statistical Analysis

All analyses were performed using R software version 4.0.2 (R Foundation, Vienna, Austria). Demographic and clinical differences between the groups were assessed using independent *t*-tests for continuous variables with normal distribution. For categorical variables, Fisher’s exact tests were employed for binary comparisons, and Chi-Square tests were used for age differences. Logistic regression, adjusted for comorbidities, was used to compare ADHD rates between subjects with and without SIgAD. Odds ratios (ORs) and their 95% confidence intervals (CIs) were calculated.

## 3. Results

### 3.1. Study Population

The study population included two groups: 772 individuals with a confirmed diagnosis of SIgAD who comprised the case group, and a 3860-individual control group without the diagnosis of SIgAD, who were matched 5:1 with the case group.

### 3.2. Demographic Characteristics of the Two Study Groups

Table 1 displays the various demographic characteristics of the two study groups. It documents a very strict matching with no real demographic differences between the cases and control groups regarding gender, mean age, age category, ethnicity group, the region in the country where the person lives, numerical SES and SES category. Details of the groups are presented in Table 1.

### 3.3. The Association between SIgAD and ADHD

As shown in Table 2, ADHD was significantly more prevalent in the SIgAD group (125 cases, 16.2%) compared to the control group without SIgAD (498 cases, 12.9%, adjusted OR 1.30, 95% CI 1.05 to 1.61, *p* = 0.017). In addition, central nervous system stimulant medications used for the treatment of ADHD were consumed more frequently by individuals in the SIgAD group than in the control group: this was found for both methylphenidate hydrochloride (2.5% vs. 1.5%, adjusted OR 1.68, 95% CI 1.00–2.82, *p* = 0.048) and methylphenidate hydrochloride slow release (4.1% vs. 2.5%, adjusted OR 1.70, 95% CI 1.13–2.55, *p* = 0.010).

### 3.4. The Associations between SIgAD and Other Morbidities

Table 2 shows the rates of other morbidities in individuals with SIgAD and the matched controls. As expected, both systemic autoimmune disease (2.6% vs. 0.9%, adjusted OR 2.98, 95% CI 1.70–5.22, *p* < 0.001) and organ-specific autoimmune diseases (5.1% vs. 1.8%, adjusted OR 2.89, 95% CI 1.94–4.31, *p* < 0.001) were significantly more prevalent in patients with SIgAD. Mood disorders were more common in patients with SIgAD than in the controls (2.1% vs. 1.2%), but the difference narrowly missed statistical significance (*p* = 0.073). The other relevant morbidities did not differ between the groups, as detailed in Table 2.

### 3.5. The Association of SIgAD with Infectious Diseases

The rates of infectious diseases in the SIgAD cases and the control group are shown in Table 3. As can be seen, the various infections were more common in the patients with SIgAD deficiency than in the matched controls, as follows: upper respiratory tract infections (26% vs. 20%, adjusted OR 1.83, 95% CI 1.48–2.28, *p* < 0.001), acute bronchitis (3.1% vs. 2.1%, adjusted OR 1.55, 95% CI 1.30–1.83, *p* < 0.001), influenza (0.9% vs. 0.6%, adjusted OR 1.65, 95% CI 1.27–2.13, *p* < 0.001), pneumonia (3.1% vs. 1.4%, adjusted OR 1.77, 95% CI 1.24–2.50, *p* < 0.001), and acute gastroenteritis (5.7% vs. 5.1%, adjusted OR 1.69, 95% CI 1.44–1.98, *p* < 0.001).

## 4. Discussion

### 4.1. New Findings

In this nationwide case–control study, we identified a novel association between SIgAD and ADHD, with ADHD being significantly more prevalent in individuals with SIgAD compared to strictly matched controls without SIgAD (adjusted OR 1.30, 95% CI 1.05 to 1.61, *p* = 0.015). Moreover, as a complementary finding, the study also documented a higher consumption of methylphenidate hydrochloride—central nervous system stimulants used to treat ADHD—among individuals with SIgAD compared to the control group (adjusted ORs 1.68 and 1.70).

### 4.2. Discussion of New Findings

The documented association between SIgAD and ADHD is in concert with previous findings and expands them. A previous study has shown that SIgAD is associated with increased rates of pediatric-onset obsessive–compulsive disorder [20], while our study extends the association to ADHD. Our study sheds further light on the growing body of evidence linking immune dysfunction to neurodevelopmental disorders [5,12,17,20]. Our finding suggests a complex interplay between immunological factors and the pathophysiology of ADHD, reinforcing previous studies on the role of inflammation in ADHD [16,21]. The observed higher prevalence of ADHD in patients with SIgAD also corresponds with the known increased risk of autoimmune disorders observed in patients with SIgAD [4,21], a relationship that has been confirmed in the present study.

The increased use of methylphenidate medications in the patients with SIgAD further supports the higher prevalence of ADHD and might suggest a potential increased severity of ADHD symptoms in this population. This finding has important clinical implications, suggesting that patients with SIgAD may require more intensive ADHD management and closer monitoring of the treatment outcomes.

Our study also corroborated previous observations that systemic and organ-specific autoimmune diseases [4] as well as respiratory and intestinal infections [1,3] were more prevalent in patients with SIgAD than in the matched controls. These are related to the immune dysfunction of SIgAD and the increased rates of various infections in patients with ADHD [9,13].

Several potential pathways may play a role in the link between SIgAD and the increased prevalence of ADHD. First, autoimmune diseases, which have an increased prevalence in patients with SIgAD, cause systemic inflammation and could contribute to neuroinflammatory processes, which have been implicated in the development of ADHD [7,22,23]. Our data revealed a notable prevalence of autoimmune diseases in the SIgAD cohort, with both systemic and organ-specific autoimmune diseases being significantly more common than in the controls. These findings underscore the potential role of autoimmunity in the SIgAD-ADHD relationship and warrant further investigation into shared pathophysiological mechanisms.

Along this line, allergic symptoms, another facet of immune hyperactivity, have been associated with ADHD [24]. In addition, alterations in neural development resulting from immune dysfunction may increase the susceptibility to ADHD, a hypothesis supported by emerging evidence of immune system involvement in neurodevelopmental disorders [11,25,26]. It is also plausible that shared genetic vulnerabilities affect both the immune and neurodevelopmental processes.

Other mechanisms relate to infectious diseases and their sequalae. As a relatively common primary immune deficiency, infectious diseases, especially respiratory and gastrointestinal, are well-established complications of SIgAD [1,3]. Infections may lead to systemic inflammation, which is associated with ADHD. Indeed, several studies have reported high levels of cytokines in individuals with ADHD, specifically interleukin (IL)-3, IL-6, and tumor necrosis factor-alpha [27,28,29]. We have found that Shigella infections during early infancy were associated with significantly increased rates of ADHD [7], probably by inducing neuroinflammations, as shown in an animal model [30]. On the other hand, we have previously documented that ADHD is associated with increased rates of diverse infectious diseases and increased use of antimicrobial agents [9].

Interestingly, the composition and diversity of the microorganisms that inhabit the human intestine (human microbiota) have been shown to influence mental health and disorders of the central nervous system [31]. Thus, the gut–brain axis signaling, wherein the gut microbiota regulates immune responses related to neuroinflammation, might also play a role in the relationship between SIgAD and ADHD. Taken together, these findings suggest that microorganisms, infection diseases, and their associated inflammation may present a plausible pathogenetic link between SIgAD and ADHD.

### 4.3. Additional Morbidities

In examining psychiatric comorbidities, we observed a trend toward an increased prevalence of mood disorders in SIgAD patients, although the increase did not reach statistical significance. Given the established comorbidity between ADHD and mood disorders in the general population [16,32], this trend merits further exploration. Anxiety disorders, while not significantly more prevalent in our SIgAD cohort, remain an important consideration in ADHD management due to their frequent co-occurrence and potential impact on treatment outcomes [16]. Interestingly, our data showed no significant differences in the prevalence of learning disabilities, adjustment disorders, or autism spectrum disorders between the SIgAD and control groups. This suggests that the immunological factors associated with SIgAD may have a more specific impact on ADHD-related neurodevelopmental pathways, rather than broadly affecting all neurodevelopmental processes. This specificity could provide insights into the underlying mechanisms linking immune functions and ADHD. This understanding might lead to new therapeutic options; for example, immunomodulatory interventions in the management of ADHD patients with concurrent immune dysfunction have been suggested [33].

### 4.4. Strengths and Limitations

#### 4.4.1. Strengths

The major strength of this study includes its nationwide, population-based, large-scale design, which encompassed all registered individuals in a large HMO; this enabled us to reach the new findings of the study. An additional strength is the strict matching algorithm used for age, gender, ethnic sector, and SES, which enhanced the reliability and generalizability of our findings.

#### 4.4.2. Limitations

The major limitation of the study is its retrospective design, as it leaves room for the possibility that unknown confounders could have influenced the results, despite our rigorous matching and adjusted analyses. The cross-sectional nature of the study limited our ability to infer causality; thus, we have used the term “association” throughout the manuscript. Prospective studies are needed to further clarify the temporal relationships and the causality between SIgAD and ADHD, and its severity.

## 5. Conclusions

The present study documents a novel association between SIgAD and ADHD, along with increased use of central nervous system stimulants in the SIgAD population. Clinicians should be aware of the higher likelihood of ADHD in patients with SIgAD, ensuring a high index of suspicion for early diagnosis and initiation of treatment. The increased prevalence of autoimmune and infectious diseases in patients with SIgAD, coupled with a significant association between SIgAD and ADHD, suggests a complex interplay between microorganisms, infections, immune function, and neurodevelopment. Future research should include exploring the shared genetic and environmental factors contributing to both SIgAD and ADHD, further investigating the role of neuroinflammation in ADHD pathogenesis, and thus examining the potential of new interventions for the treatment and management of ADHD, such as immunomodulatory interventions.

## Figures and Tables

**Table 1:** Demographic characteristics of the subjects with selective IgA deficiency and the matched controls.

Variable		IgA Deficiency	Controls	*p* Value	Odds Ratio
N		772	3860		
Gender	Female	386 (50.0%)	1930 (50.0%)	1	1
	Male	386 (50.0%)	1930 (50.0%)	1	1
Age, mean ± SD, years		22.0 ± 17.5	22.2 ± 17.7	0.88	
Age category, years	≤9	200 (25.91%)	1000 (25.91%)	1	1
	10–18	258 (33.42%)	1290 (33.42%)	1	1
	19–29	126 (16.32%)	630 (16.32%)	1	1
	30–39	62 (8.03%)	310 (8.03%)	1	1
	40–49	42 (5.44%)	210 (5.44%)	1	1
	50–59	46 (5.96%)	230 (5.96%)	1	1
	60–69	21 (2.72%)	105 (2.72%)	1	1
	70–79	11 (1.42%)	55 (1.42%)	1	1
	80–89	6 (0.78%)	30 (0.78%)	1	1
Ethnic sector	Arab	79 (10.2%)	395 (10.2%)	1	1
	General	397 (51.4%)	1985 (51.4%)	1	1
	Jewish Ultra-orthodox	296 (38.3%)	1480 (38.3%)	1	1
Region in the country	Center	234 (30.3%)	911 (23.6%)		
	Jerusalem	269 (34.8%)	1332 (34.5%)		
	North	117 (15.2%)	633 (16.4%)		
	South	152 (19.7%)	984 (25.5%)		
Socioeconomic status (1–10)		4.80 ± 1.88	4.73 ± 1.87	0.3284	
	~missing~	2 (0.26%)	10 (0.26%)		
Socioeconomic status category	1–3 Low	244 (31.69%)	1220 (31.69%)	1	1
	4–6 Middle	380 (49.35%)	1900 (49.35%)	1	1
	7–10 Middle–Upper	146 (18.96%)	730 (18.96%)	1	1

**Table 2:** Prevalence of ADHD, other relevant morbidities, and methylphenidate consumption in subjects with selective IgA deficiency and matched controls.

Condition Number (%)	SIgA Deficiency (Cases) (*n* = 772)	Control Subjects (*n* = 3860)	*p*-Value	Adjusted Odds Ratio * (95% CI)
ADHD	125 (16.2%)	498 (12.9%)	0.017	1.30 (1.05–1.61)
Other morbidities				
Anxiety disorders	31 (4.0%)	134 (3.5%)	0.456	1.16 (0.78–1.72)
Adjustment disorders	4 (0.5%)	20 (0.5%)	0.989	1.00 (0.34–2.93)
Mood disorders	16 (2.1%)	48 (1.2%)	0.073	1.69 (0.95–2.99)
Autism spectrum disorder	3 (0.4%)	16 (0.4%)	0.912	0.94 (0.27–3.23)
Depression	5 (0.6%)	29 (0.8%)	0.187	0.86 (0.59–1/13)
Learning disabilities	33 (4.3%)	166 (4.3%)	0.975	0.99 (0.68–1.46)
Autoimmune diseases				
Systemic autoimmune diseases	20 (2.6%)	34 (0.9%)	<0.001	2.98 (1.70–5.22)
Organ-specific autoimmune diseases	39 (5.1%)	70 (1.8%)	<0.001	2.89 (1.94–4.31)
Asthma	72 (9.3%)	322 (8.3%)	0.359	1.13 (0.85–1.48)
Diabetes mellitus	32 (4.1%)	112 (2.9%)	0.087	1.46 (0.95–2.20)
Fibromyalgia	8 (1.0%)	26 (0.7%)	0.255	1.55(0.79–2.45)
Medication use				
Methylphenidate (immediate release)	19 (2.5%)	57 (1.5%)	0.048	1.68 (1.00–2.82)
Methylphenidate (slow release)	32 (4.1%)	95 (2.5%)	0.010	1.70 (1.13–2.55)

* Multiple regression models were used to adjust for systemic and organ-specific autoimmune comorbidity.

**Table 3:** Rates of various infections in subjects with selective IgA deficiency and matched controls by regression analysis.

Infection Number (%)	SIgA Deficiency (Cases) (*n* = 772)	Control Subjects (*n* = 3860)	Adjusted Odds Ratio (95% CI)	*p*-Value
Influenza	7 (0.9%)	22 (0.6%)	1.65 (1.27–2.13)	<0.001
Upper respiratory tract infections	198 (26%)	756 (20%)	1.83 (1.48–2.28)	<0.001
Acute bronchitis	24 (3.1%)	81 (2.1%)	1.55 (1.30–1.83)	<0.001
Gastroenteritis	44 (5.7%)	198 (5.1%)	1.69 (1.44–1.98)	<0.001
Pneumonia	24 (3.1%)	54 (1.4%)	1.77 (1.24–2.50)	<0.001

## Data Availability

The datasets generated during and/or analyzed during the current study are available from the corresponding author and Ariel Israel on reasonable request.
